# Unpaid leave on COVID-19: The impact of psychological breach contract on emotional exhaustion: The mediating role of job distrust and insecurity

**DOI:** 10.3389/fpsyg.2022.953454

**Published:** 2022-07-22

**Authors:** Syed Usman Qadri, Mingxing Li, Zhiqiang Ma, Safwan Qadri, Chengang Ye, Muhammad Usman

**Affiliations:** ^1^School of Management, Jiangsu University, Zhenjiang, China; ^2^Department of Public Administration, Wuhan University, Wuhan, China; ^3^Business School, University of International Business and Economics, Beijing, China; ^4^Department of Management Science, Iqra National University, Peshawar, Pakistan

**Keywords:** COVID-19, psychological breach contract, emotional exhaustion, job distrust, job insecurity

## Abstract

This research intends to increase awareness of the existence of psychological breach contracts on emotional exhaustion in the context of a prolonged COVID-19 pandemic, with the function of organizational distrust (OD) and job insecurity (JI) serving as mediating factors. We used partial least squares structural equation modeling (PLS-SEM) to look at the 437 questionnaires that private sector workers in Pakistan filled out during the COVID-19 outbreak. The findings of direct and indirect effects show that (PBC) psychological breach contract directly leads to emotional exhaustion (EH) and has a significant indirect relationship through job insecurity (JI). Moreover, psychological contract breach (PBC) directly leads to emotional exhaustion (EH) and has a significant indirect relationship through organization distrust (OD). The study shows both theoretical and practical implications, as well as areas where more research needs to be done.

## Introduction

After COVID-19, the world was hit by a terrible and unexpected crisis that had a big impact on every part of life. Some of the things that were done to stop the spread of the epidemic were a lockdown and isolating people from each other, both of which led to the collapse of the company (Karim et al., 2020; Fernandes, 2020). The closing of industries caused a lot of bad things to happen at work, like people quitting on their own, being suspended for a short time, having their hours cut back, and taking paid or unpaid leave (Gössling et al., 2020). Ali and Çobanoglu (2020) admit that businesses in the tourism, transportation, and hospitality industries were hit hard. Foo et al. (2020) also confirmed that thousands of people in the various industries lost their employment or were forced on unpaid leave. Most airlines, like Cathay Pacific and Singapore Airlines, sent their employees on leave without pay. ILO (2020) argued that more than 25,000 workers, or a mandatory unpaid leave of 75% of all employees, should be covered by this policy. Aside from the airline industry, the pandemic caused a lot of trouble in the service industries. Because there weren’t enough customers, about 23,000 Vietnamese workers had to take short vacations. 340 people who worked at restaurants in New Zealand had to be fired (ILO, 2020). The unpaid vacation made the employee angry, which made the employee’s breach of the psychological contract worse. Research shows that PBC has a big effect on how productive employees are and how likely they are to leave their jobs. Additionally, Zanzibar’s labor rules enable flexibility for businesses to lay off their employees without liability if they allow unpaid leave for a month, and after this time period, an additional 3 months. Two conditions must be met for a corporation to be permitted to lay off its employees, as stipulated by the labor laws. Firstly, when a business is unable to pay its personnel owing to economic difficulties, Additionally, should there be a force majeure, such as a pandemic, things will be delayed until the force majeure has passed. As a result of the outbreak, hotels, tour operators, and restaurant owners placed their personnel on many months of unpaid leave. Hence, it is necessary to investigate how such psychological contract breaches can result in organizational distrust, job insecurity (JI), and emotional exhaustion (EH).

Research on PBC previously focused on the connection between the PBC and the identity of the organization, engagement and loyalty in the workplace, and performance at work (Li et al., 2016). Chen and Wu (2017) did an analysis of the PBC’s role as a mediator in the relationship between leadership styles and the wish to leave the organization. PBC has a direct impact on the feeling of happiness and accomplishment (Ampofo, 2020). Likewise, the study of Karatepe et al. (2020) also focused on increasing intention to work and engagement in proactive activity.

Therefore, related studies provide a clear knowledge of the effect of PBC on employee distrust (ED) and turnover intentions. However, the studies on PBC and the COVID-19 pandemic condition are quite rare. In this regard, this research aims to improve the awareness of the existence and impact of psychological breach contract (PBC) on emotional exhaustion (EH) in the situation of a prolonged COVID-19 pandemic, with a mediating role of organization distrust (OD) and job insecurity (JI). The main goal of the study was to check on the PBC during the COVID-19 pandemic in the private sector of Pakistan. As far as we know, this pandemic affects all sectors, but in Pakistan, the private sector employees are hit the hardest. They are instructed to either take unpaid leave or leave the company. That makes them feel very bad about the company. Pakistan is a developing nation where there are no explicit norms and regulations governing employee rights. Therefore, this study is useful for determining whether companies violated the psychological contract with their employees during the COVID-19 pandemic. In such a precarious financial state, it is extremely difficult for enterprises in developing economies to pay full wages. Even in the developed world, many businesses give unpaid leave to their employees. The study is significant from both a theoretical and practical standpoint. Theoretically, it confirms whether private sector employees were affected by the COVID-19 epidemic and whether they considered there was a psychological breach of contract. In practice, the government may have the chance to address this issue among private sector employees and provide them with economic and psychological help. In the meantime, the government might be able to set rules and guidelines for unpaid leave in the private sector. The study has the following research objectives:

• To identify the impact of PBC on emotional exhaustion in the COVID-19 Pandemic.
• To study the impact of PBC on emotional exhaustion with the mediation role of organization distrust (ID) and job insecurity (JI) in the COVID-19 Pandemic.

The remaining sections of this work are organized as follows. In the section titled “Related literature and hypothesis development,” we create a conceptual model of the study and related literature. Measurements and data gathering are contained in the “Materials and methods” section. The Results and Findings section contains the study’s analysis. In addition, the discussion section contains a discussion of the results, study limitations, and imperial implications. Finally, the last section gives the study’s conclusion.

## Related literature and hypothesis development

### Perceived contract breach and employees outcomes

Contemporary organizations need to be aware of their employees’ attitudes and behaviors to figure out PBC. Everyone knows that the PBC depends on the rule of reciprocity. Both the employees’ and the employer’s hopes are met, which creates a connection between the two. In today’s world, organizations have fewer resources and are under more pressure to stay profitable and competitive (Arunachalam, 2020). Individuals expect a return on their contributions to the organization as a result (Balabanova et al., 2019). PBC is always higher when an employer doesn’t do what they said they would do, either directly or indirectly (Arunachalam, 2020). PBC is when an organization doesn’t live up to some of the promises it made about an employment arrangement, either directly or indirectly (Morrison and Robinson, 1997). Li and Chen (2018) and Shen et al. (2019) found that studying the PBC can lead to a number of inappropriate and adverse outcomes, such as absenteeism, counterproductive work behavior, intention to leave, and stress, which is not good for organizations. Other studies have shown that when people are exposed to the PBC, they may experience anxiety, depression, exhaustion, and a sense of injustice.

Using social exchange theory (SET; Blau, 1964) as a basic framework, the current study examines how lack of reciprocity may occur in the setting of JI and OD. SET is one of the most popular ways to think about how employees react in organizations. A social exchange consists of a sequence of encounters between an employer and an employee that establishes mutual obligations. Most people think that these interactions depend on and are affected by what other people do (Cropanzano and Mitchell, 2005). These transactions may involve activities, material or intangible resources, as well as social and economic repercussions. Employer-employee relationships can be conceptualized as an exchange of benefits, costs, resources, and outcomes. So, we can use this framework to understand the relationship between employer and employee. Fundamental to social exchange theory is the notion that an individual develops a social relationship on the basis of mutually advantageous partnerships (Qadri et al., 2022).

According to Greenhalgh and Rosenblatt (1984, p. 438), job insecurity (JI) is the “perceived inability to sustain desired continuity in a threatened job position.” In particular, those who experience insecurity fear losing their jobs. The Job Insecurity Index (JI) might be considered a source of resource loss because it indicates the fear of losing a specific job and the psychological implications of this subjective experience. Especially when an employer doesn’t do what they promised to do for their employees, it may show that the employee isn’t important to the employer and make the employee feel less safe about their job (Cavanaugh and Noe, 1999; Restubog et al., 2006). Furthermore, seeing PBC as a cause of job insecurity is similar to the COR’s (Hobfoll, 2001) view that people are more likely to lose resources again if they have lost them once.

H1: PBC is positively related to job insecurity (JI).

When a psychological contract is broken, the continuous cycle of giving and receiving is broken, and the employee’s loyalty to the company and its goals is diverted (Cassar and Briner, 2011). Cassar and Briner (2011) concluded that people are willing to put in a lot of work because they believe that organizations will keep their promises. This has created a psychological contract that keeps the cycle going. It is commonly recognized that when employees perceive a breach of a psychological contract, they respond negatively. This makes people think that their companies don’t have integrity, which makes people feel bad about them (Sarikaya and Bayrak Kök, 2017). Employees are reported to feel dissatisfied upon discovering a breach of the psychological contract, therefore decreasing their organizational commitment and performance over time (Bunderson, 2001). Therefore, if the psychological contract is violated, employees may lose faith in the organization. Consequently, the following hypothesis is put forward:

H2: PBC is positively related to employees’ distrust toward organizations.

The employee-employer relationship is best described by the psychological contract theory (Reyes Flores et al., 2019). On the other hand, employees experience a psychological contract breakdown (Morrison and Robinson, 1997) when their companies don’t meet what they think are mutual promises. This causes stress and, eventually, work strain (Gakovic and Tetrick, 2003). Russell and Mehrabian (1974) established that a person’s body, or internal state, reflects and responds to information about their environment. Any changes that a company wants to make to how its workers do their jobs could be the stimulus (Goi et al., 2018). The PBC was a good forecaster of how workers would feel, so the breach will make workers feel bad (Piccoli and De Witte, 2015). As a result, we proposed this point.

H3: Employees’ PBC is positively related to their emotional exhaustion (EH).

Poor job performance, job satisfaction, psychological wellbeing, and employee turnover are all linked to job instability (Cheng and Chan, 2008; Staufenbiel and König, 2010; De Witte et al., 2016). According to the COR hypothesis (Hobfoll, 1989), a person may become emotionally exhausted if they perceive a threat to their resources and do not receive sufficient gains in return. During the COVID-19 pandemic, restaurant frontline workers’ perceptions of JI could be a risk to their resource loss and increase their EH, since losing their jobs could lead to bad things like depression (Kessler and Walters, 1998). As a result, the following hypothesis has been proposed:

H4: Job insecurity (JI) is positively related to emotional exhaustion (EH).

Because of its significance in evoking good employee reactions and behaviors at work, trust between an employee and their supervisor (DeConinck, 2010; Jiang et al., 2019) has long been a significant problem in the modern world. Similarly, employees have a level of trust in their bosses, coworkers, and the organization/company as a whole (Susskind et al., 2003; DeConinck, 2010). Supervisors and coworkers are social partners for employees at work (Susskind et al., 2003; Loi et al., 2014).

Employees who are hated and feared should experience EH for a variety of reasons. Low levels of trust may amplify stress, resulting in a greater level of EH (Maslach et al., 2001; Halbesleben and Buckley, 2004). Lewicki and Bunker (1996) say that building trust leads to reciprocity, which lowers EH in the workplace and vice versa.

H5: Organization distrust (OD) is positively related to employees’ emotional exhaustion (EH).

### Conceptual model of the study

**FIGURE 1:**
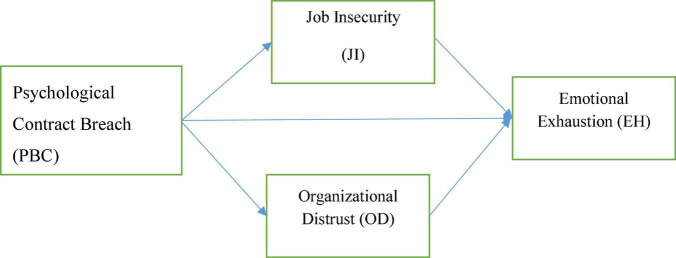
Conceptual model of the study.

Considering the above hypotheses (H1, H2, H4, and H5), we further suggested that job insecurity (JI) and distrust (JD) will serve as mediating mechanisms that interpret the independent variable impact (PBC) on the outcome (emotional exhaustion) as shown in Figure 1. When a company doesn’t do what it says it will do for its employees, it may mean that the company doesn’t value the employee. Because of this, it’s more likely that people’s sense of security in their jobs and relationships with their companies will go down (Cavanaugh and Noe, 1999; Restubog et al., 2006) and will continue to worsen, which will result in a decline in trust placed in the organizations. The COR’s (Hobfoll, 2001) idea that losing a resource once makes people more sensitive to losing it again is consistent with the idea that PBC is a cause of insecurity. We say that an initial loss from PBC leads to a second loss in the form of job insecurity for the employee (JI). On the other hand, treating PBC as a source of distrust is consistent with SET (Blau, 1964), which believes in reciprocal relationships between an employer and employees. Studies in the extant literature have evidenced that trust in organizational factors has a significant association with major employee affective and cognitive responses such as burnout, wellbeing, job satisfaction, psychological distress, mental health, and commitment (see for example; Loi et al., 2014; Karatepe and Olugbade, 2017). Hence, the following hypotheses can be derived from the foregoing research and theoretical reasoning.

H6: Job insecurity (JI) mediates the relationship between psychological contract breach (PBC) and emotional exhaustion (EH).
H7: Organization distrust (OD) mediates the relationship between psychological contract breach (PBC) and emotional exhaustion (EH).

## Material and methods

### Measure

This study used all previous related measures. Five elements from Robinson and Morrison (2000) list of psychological contract breaches (PBC) were adopted. Five items from Moore’s (2000) study were used to measure emotional exhaustion (EH). JI was measured in the study of Karatepe (2013). In addition, the items of OD were adapted from Adams et al. (2010). Each question was scored on a Likert scale ranging from 1 to 5, with 1 indicating strong disagreement and 5 indicating strong agreement. For the purpose of determining the content validity, three experts were consulted: three from the industry and three from academia. Some of the elements were changed as a result of expert recommendations. The screening questions were designed to identify only those employees who were on unpaid leave from the department and industry. In the end, 30 employees took part in a pilot study. According to Cronbach alpha, the instrument’s reliability was tested at greater than 0.70.

### Data collection

In order to get information from Pakistani employees, we carried out a cross-sectional survey. The researchers asked people to take part in their studies using two methods: snowball sampling and purposeful sampling. The sample was taken from people who work in the private sector in Pakistan. Because of the state’s influence, employees in the government sector rarely face pay problems. Therefore, only personnel from the private sector were included in the final analysis. Respondents were assured that they would remain anonymous and that they could choose whether or not to take part in the study. The questionnaire was distributed online to respondents in September 2020. And only those respondents who responded by 30 November 2020 were considered final. After removing missing data and answers from people who didn’t care, 437 questionnaires were left that could be used to further study. This sample size satisfies the requirements for the needed sample size (Ali et al., 2021). Based on the demographic information, 58.6% of the respondents were men. In comparison, 41.4% of respondents were women, 62.7% were between the ages of 19 and 26, 28.8% had a master’s degree, 88.6% were from cities, and 305 respondents were single as shown in Table 1.

**TABLE 1:** Demographic.

Variable	Category	Distribution	
		
		Frequency	Percentage
Age	19–26	274	62.7
	>26–33	91	20.8
	>33–40	46	10.5
	>40–47	6	1.4
	>47–54	11	2.5
	>54–61	7	1.5
	>61	2	0.5
Gender	Male	256	58.6
	Female	181	41.4
Education	No formal education	6	1.4
	Primary (year 5)	3	0.7
	Secondary school certificate (year 10)	2	0.4
	Higher secondary school certificate (year 12)	20	4.6
	Undergraduate (year 16)	116	26.6
	Masters (year 18)	126	28.8
	MBBS or BDS	133	30.4
	DVM	12	2.7
	BE	3	0.7
	Others	16	3.7
Income *^a^*	45,000–55,000	177	40.5
	>55,000–65,000	36	8.2
	>65,000–75,000	79	18.1
	>75,000–85,000	30	6.9
	>85,000–95,000	32	7.3
	>95,000–105,000	27	6.2
	>105,000	56	12.8
Location	City	387	88.6
	Suburb	16	3.7
	Village	34	7.8
Marital status	Single	305	69.8
	Married	117	26.8
	Divorced	12	2.7
	Widowed	3	0.7

^a^Income is given in Pak Rupee (exchange rate: USD 1 = 165 PKR).

## Results and findings

Constructs and underlying dimensions were verified using a principal component analysis (PCA) in SPSS 24.0 (Costello and Osborne, 2005). Several ways were used to look at the PCA results (Kaiser, 1974; Field, 2017). The results showed a good KMO value (KMO = 0.91), and the Bartlett test was statistically significant (*p* < 0.05). In light of these findings, we can proceed with further component analysis because there is a strong correlation between samples and items (Kaiser and Rice, 1974). From 0.527 to 0.777, the communalities show that each item had a good amount of shared variation. Eleven parts were taken out based on the root mean (Eigen value) criteria. These 11 factors explained 52.99% of the total variation, which is more than the minimum criteria, or >50%, and is therefore acceptable (Beavers et al., 2013). In the same way, each individual component explained between 4.23 and 12.77% of the difference. This meant that each factor contributed the same amount to the total variance explained and that no single factor could have dominated the total variance explained (i.e., more than 50%) or caused common method bias (Podsakoff et al., 2003).

For further analysis, each item had to be looked at very closely. Things that had a factor lead of less than 0.40 were immediately taken out of the analysis (Hair, 2010). To get rid of things with low factor loading or cross-loading, an iterative process was used. Items were kept in the final round if their factor loadings were between 0.712 and 0.869. Table 2 gives a brief overview of what PCA found.

**TABLE 2:** EFA, composite reliability, and AVEs.

Constructs	Items	Standardized loadings	AVE	α	CR
Psychological contract breach			0.700	0.785	0.875
	PCB 1	0.834			
	PCB 2	0.869			
	PCB 3	0.806			
Job insecurity			0.661	0.745	0.854
	JI 1	0.795			
	JI 2	0.832			
	JI 3	0.811			
Organizational distrust			0.692	0.779	0.871
	OD 1	0.801			
	OD 2	0.825			
	OD 3	0.869			
Emotional exhaustion			0.618	0.796	0.866
	EE 1	0.801			
	EE 2	0.859			
	EE 3	0.766			
	EE 4	0.712			
KMO measure of sampling adequacy			0.910		
Bartlett’s test			0.000		
Total variance explained			52.99%		

### Convergent validity and discriminant validity of constructs

Factor loading (Wixom and Watson, 2001), composite reliability (Nunnally, 1978; Burns et al., 2016), and average variance extracted (Fornell and Larcker, 1981) were used to figure out the convergent validity of the measures, while the HTMT ratio was used to figure out the discriminant validity (Henseler et al., 2015). The AVEs ranged from 0.618 to 700, and the CR ranged from 0.854 to 0.875, all of which met the predetermined criteria (i.e., λ ≥ 0.4, AVEs ≥ 0.5, and CR ≥ 0.7).

### Structural model analyses

The structural model hypotheses were tested using partial least squares structural equation modeling (PLS-SEM) with Smart PLS v.3.0. PLS-SEM is known for being mostly flexible, but strict when it comes to getting reliable and valid results (Sarstedt et al., 2016). PLS SEM is a great way to look at data in many business and management studies because it has a high statistical power when the data isn’t normally distributed and is more tolerant of the data assumptions of heteroscedasticity (Hair et al., 2016). Also, PLS-SEM is a better way to test complex and highly intertwined relationships with multiple direct and indirect effects in a population of car buyers who may not be spread out in the same way. For this study, this is important.

Before the main analysis of estimating direct and indirect paths, the results showed that there was no multicollinearity among the latent constructs because the variance inflation factor (VIF) scores for the latent constructs were well below the cut-off value of 10 (Hair, 2010). The bootstrap method was used to do a path analysis with 5,000 bootstrapped samples and 95% bias-corrected bootstrap intervals (Hair et al., 2016). In the sections that follow, both direct and indirect estimates are shown in Table 3.

**TABLE 3:** Model 1a: Direct, indirect, and total effects.

Predictor (X)	Mediator (M)	Dependent variable (Y)	Direct effect	Indirect effect	Total effect	*p*-value	*R* ^2^	*f* ^2^	*Q* ^2^
Psychological contract breach	Job insecurity	Emotional exhaustion	0.385 *(0.291, 0.466)*	0.198 *(0.142, 0.258)*	0.583	0.000	0.459	0.207	0.192
	Organizational distrust		0.401 *(0.301, 0.495)*	0.143 *(0.098, 0.258)*	0.544	0.000	0.698	0.224	0.204

Values in parenthesis and italics in direct and indirect effects columns are lower and upper bound bias-corrected confidence intervals of the estimates obtained as a results of bootstrapping procedure.

### Model fit indices

Covariance-based structural equation modeling (CB-SEM) makes it easy to test model fit indices to figure out how well a model works. This is not the case with Smart PLS (Hair et al., 2016). You can get a good idea of how good the model is by finding out how it compares to others. Model fit indices can be used with R2 values to find out how strong a model is and how well it predicts. Model fit indices like standardized root mean square residual (SRMR) and root mean square theta (RMS-theta) have been used to measure how good an ESCCB model based on VBN theory is. The SRMR is the difference between the correlation that was seen and the correlation that was predicted by the model (Hair et al., 2016). As an absolute measure of how well the model fits, it uses the average number of differences between the correlation that was seen and the correlation that was expected. SRMR helps find and fix any mistakes in the specifications of the model (Hair et al., 2016). The SRMR value of a model that fits well is 0.10 or 0.08 (Hair et al., 2017). RMS-theta is a way to measure the root mean squared residual covariance matrix of the outer model error terms (Lohmöller, 1989). This measure has been used to check how well a model fits because it is suggested when the model only has reflective measures (Hair et al., 2016). RMS-theta should be close to zero, and it is thought that a good model has a value of 0.12 or less (Hair et al., 2016). The results show that PBC explains 45.9% of the difference in EH through JI and 69.8% through organization distrust.

### Estimates of direct effects and indirect effects

The estimates of direct and indirect effects show that PBC directly leads emotional exhaustion (EH) (β = 0.38, *p* < 0.05) and has a significant indirect relationship through job insecurity (JI) (β = 0.198, *p* < 0.05). On the other hand, PBC directly leads emotional exhaustion (EH) (β = 0.401, *p* < 0.05) and has a significant indirect relationship through OD (β = 0.143, *p* < 0.05) as shown in Table 4.

**TABLE 4:** Model 1b: Intercorrelations, variance inflation factor (VIF), and discriminant validity of the constructs.

Constructs	VIF	A	B	C	D
Psychological contract breach	1.599, 1.519	*0.836*			
Job insecurity	1.514, 1.456	(0.687) 0.530**	*0.813*		
Organizational distrust	1.368, 1.291	(0.554) 0.435**	(0.514) 0.392**	*0.832*	
Emotional exhaustion	1.535	(0.355) 0.290**	(0.364) 0.289**	(0.328) 0.266**	*0.786*

** Correlations significant at p < 0.01. Values in parenthesis and italics in direct and indirect effects columns are lower and upper bound bias-corrected confidence intervals of the estimates obtained as a results of bootstrapping procedure

## Discussion

The goal of this study was to find out how unpaid leaves during COVID-19 affected emotional exhaustion (EH) directly and indirectly through job insecurity (JI) and distrust of the organization (OD). As a result, we generated seven hypothesis. The H1 and H2 hypotheses were supported, indicating a positive relationship between PBC and JI and OD. The findings indicated that the PBC exacerbated their intention toward employees’ job insecurity and their thought regarding OD as a result of the unpaid leave granted in COVID-19. The earlier research are confirmed the same results, indicating the PBC increases negative related to JI and OD (Piccoli and De Witte, 2015; Costa et al., 2017). Our hypothesis H3, H4 and H5 are confirming that PBC, JI and OD is positively related to EH. These relations were in line with what was found in earlier research on OD and plans to leave (Kim and Yang, 2017; Sarikaya and Bayrak Kök, 2017; Kang et al., 2018). Moreover,

Hypothesis H6, H7 are also confirming that Job insecurity (JI) and organization distrust (OD) mediate the relationship between PBC and employees EH during the COVID-19 Pandemic. These findings confirming the prior research (Loi et al., 2014; Karatepe and Olugbade, 2017). Organizational mistrust resulting from a breach of the psychological contract may indicate that employees expected management to make better judgments to protect them and to react more effectively. Guo et al. (2017) showed that managers’ decisions could cause distrust in an organization. During the 2011 political crisis in Egypt, the vast majority of tourism industry employees planned to abandon their jobs. They stopped believing in their organization because their uncertain situation made it impossible for them to do their jobs (Elshaer and Saad, 2017).

### Limitation and future direction

This study has certain limitations and proposes some future directions. First, this study only looked at people who work in the private sector. In the future, researchers may get data from other government and semi-government employees to get a better idea of how things work in general and make the results more general. Second, many other psychological factors may also have an effect during a pandemic. Further studies could also be conducted to compare the PBC with other emotional factors to figure out why people take unpaid leaves. Lastly, this study only looked at data from the COVID-19 pandemic. More research could be done during different political and economic crises to confirm the results in a more generalize way.

### Practical implications

The current study has many implications for managers in the real world. The main point of this study is that organizations need to be proactive about managing the psychological contracts of their employees. This is because employees’ perceptions of unfulfilled organizational promises can lead to OD, JI, and EH. To this end, prior studies suggest several measures that both can either prevent or mitigate the negative consequences of organizational contract breaches (e.g., Chih et al., 2016). For instance, providing authentic information during recruitment process, career development, making realistic promises, and helping managers to effectively manage and understand employees organizational promises perceptions. Next, the study findings suggests two important underlying mechanisms i.e., feeling of JI and employees distrust between perception of PBC and EH. These findings reveals that when employees experiencing contract breaches, employees are likely to feel more insecure about their employment and also feeling of distrust on their management may be observed. Consequently, employees turn to be feel more exhausted in such situations. Hence, managers should be more sensitive to the promises been made with their employees. It might help to take more remedial actions to reduce the effects of broken contracts on JI, OD, and EH. For example, managers can ask for an honest apology or a clear explanation of why a contract was broken, and they can also bring up any other opportunities and work continuity. These kinds of explanations might help employees feel less upset about psychological contract breaches (Morrison and Robinson, 1997).

## Conclusion

This present study emphasized on the important role of employee’s perception of psychological contract breaches on emotional exhaustion via job insecurity and distrust. The findings suggest that PBC positively predict negative outcomes such as job insecurity (JI), employees distrust (JD), and emotional exhaustion (EH). Further, the results confirm the important role of two underlying mechanisms i.e., JI and distrust between PBC and employees emotional exhaustion (EH). These findings further enrich the literature on employees PBC, and suggest managers numerous ways to mitigate its impact on their workers in the organizations. Future, research scholars may conduct other researches in their own cultural context to check whether they could observe the similar results or not.

## Data availability statement

The raw data supporting the conclusions of this article will be made available by the authors, without undue reservation.

## Ethics statement

Ethical review and approval was not required for the study on human participants in accordance with the local legislation and institutional requirements. The patients/participants provided their written informed consent to participate in this study.

## Author contributions

SUQ, ML, and ZM contributed to the conception and design of the study. SQ and MU organized the database. SQ performed the statistical analysis. CY wrote the first draft of the manuscript. SUQ, ML, ZM, CY, SQ, and MU wrote sections of the manuscript. All authors contributed to manuscript, read, and approved the submitted version.

## Conflict of interest

The authors declare that the research was conducted in the absence of any commercial or financial relationships that could be construed as a potential conflict of interest.

## Publisher’s note

All claims expressed in this article are solely those of the authors and do not necessarily represent those of their affiliated organizations, or those of the publisher, the editors and the reviewers. Any product that may be evaluated in this article, or claim that may be made by its manufacturer, is not guaranteed or endorsed by the publisher.

## References

[B1] Adams J. E. Highhouse S. Zickar M. J. (2010). Understanding general distrust of corporations. *Corp. Reput. Rev.* 13 38–51. 10.1057/crr.2010.6

[B2] Ali F. Ciftci O. Nanu L. Cobanoglu C. Ryu K. (2021). Response rates in hospitality research: an overview of current practice and suggestions for future research. *Cornell Hosp. Q.* 62 105–120. 10.1177/1938965520943094

[B3] Ali F. Çobanoglu C. (2020). *Global Tourism Industry may Shrink by More than 50% due to the Pandemic.* Melbourne, VIC: The conversation.

[B4] Ampofo E. T. (2020). Do job satisfaction and work engagement mediate the effects of psychological contract breach and abusive supervision on hotel employees’ life satisfaction? *J. Hosp. Mark. Manage.* 30 282–304. 10.1080/19368623.2020.1817222

[B5] Arunachalam T. (2020). The interplay of psychological contract breach, stress and job outcomes during organizational restructuring. *Ind. Commer. Train.* 53 15–28. 10.1108/ICT-03-2020-0026

[B6] Balabanova E. Ehrnrooth M. Koveshnikov A. Efendiev A. (2019). Employee exit and constructive voice as behavioral responses to psychological contract breach in Finland and Russia: a within-and between-culture examination. *Int. J. Hum. Resour. Manage.* 33 360–391. 10.1080/09585192.2019.1699144

[B7] Beavers A. S. Lounsbury J. W. Richards J. K. Huck S. W. Skolits G. J. Esquivel S. L. (2013). Practical considerations for using exploratory factor analysis in educational research practical assessment. *Res. Eval.* 18:6.

[B8] Blau P. M. (1964). Social exchange theory. 3:62.

[B9] Bunderson J. S. (2001). How work ideologies shape the psychological contracts of professional employees: doctors’ responses to perceived breach. *J. Organ. Behav.* 22 717–741. 10.1002/job.112

[B10] Burns A. C. Veeck A. Bush R. F. (2016). *Marketing Research.* London: Pearson Educ.

[B11] Cassar V. Briner R. B. (2011). The relationship between psychological contract breach and organizational commitment: exchange imbalance as a moderator of the mediating role of violation. *J. Vocat. Behav.* 78 283–289. 10.1016/j.jvb.2010.09.007

[B12] Cavanaugh M. A. Noe R. A. (1999). Antecedents and consequences of relational components of the new psychological contract. *J. Organ. Behav.* 20 323–340. 10.1002/(SICI)1099-1379(199905)20:3<323::AID-JOB901>3.0.CO;2-M

[B13] Cheng G. H. L. Chan D. K. S. (2008). Who suffers more from job insecurity? A meta-analytic review. *Appl. Psychol.* 57 272–303. 10.1111/j.1464-0597.2007.00312.x

[B14] Chen T.-J. Wu C.-M. (2017). Improving the turnover intention of tourist hotel employees: transformational leadership, leader-member exchange, and psychological contract breach. *Int. J. Contemp. Hosp. Manage.* 29 1914–1936. 10.1108/IJCHM-09-2015-0490

[B15] Chih Y.-Y. Kiazad K. Zhou L. Capezio A. Li M. Restubog S. L. D. (2016). Investigating employee turnover in the construction industry: a psychological contract perspective. *J. Constr. Eng. Manage.* 142:04016006. 10.1061/(ASCE)CO.1943-7862.0001101 29515898

[B16] Costa S. P. Coyle-Shapiro J. A. Neves P. (2017). “Psychological contract breach and its correlates: effects of culture and country level factors,” in *Academy of Management Proceedings*, Vol. 2017 (Briarcliff Manor, NY: Academy of Management), 13817.

[B17] Costello A. B. Osborne J. W. (2005). Best practices in exploratory factor analysis: four recommendations for getting the most from your analysis. *Pract. Assess. Res. Eval.* 10 1–9.

[B18] Cropanzano R. Mitchell M. S. (2005). Social exchange theory: an interdisciplinary review. *J. Manage.* 31 874–900. 10.1177/0149206305279602

[B19] De Witte H. Pienaar J. De Cuyper N. (2016). Review of 30 years of longitudinal studies on the association between job insecurity and health and well-being: is there causal evidence? *Aust. Psychol.* 51 18–31. 10.1111/ap.12176

[B20] DeConinck J. B. (2010). The effect of organizational justice, perceived organizational support, and perceived supervisor support on marketing employees’ level of trust. *J. Bus. Res.* 63 1349–1355. 10.1016/j.jbusres.2010.01.003

[B21] Elshaer I. A. Saad S. K. (2017). Political instability and tourism in Egypt: exploring survivors’ attitudes after downsizing. *J. Policy Res. Tour. Leis. Events* 9 3–22. 10.1080/19407963.2016.1233109

[B22] Fernandes N. (2020). *Economic Effects of Coronavirus Outbreak (COVID-19) on the World Economy.* Available online at: https://ssrn.com/abstract1/43557504 (accessed April 3, 2020).

[B23] Field A. (2017). *Discovering Statistics Using IBM SPSS Statistics: North American Edition.* Thousand Oaks, CA: SAGE Publications.

[B24] Flores G. R. Guaderrama A. I. M. Arroyo J. C. Gómez J. A. H. (2019). Psychological contract, exhaustion, and cynicism of the employee: its effect on operational staff turnover in the northern Mexican border. *Contaduría Adm.* 64 1–19.

[B25] Foo L. P. Chin M. Y. Tan K. L. Phuah K. T. (2020). The impact of COVID-19 on tourism industry in Malaysia. *Curr. Issues Tour.* 24 2735–2739.

[B26] Fornell C. Larcker D. F. (1981). Evaluating structural equation models with unobservable variables and measurement error. *J. Mark. Res.* 18 39–50. 10.2307/3151312

[B27] Gakovic A. Tetrick L. E. (2003). Psychological contract breach as a source of strain for employees. *J. Bus. Psychol.* 18 235–246. 10.1023/A:1027301232116

[B28] Goi M.-T. Kalidas V. Yunus N. (2018). Mediating roles of emotion and experience in the stimulus-organism-response framework in higher education institutions. *J. Mark. High. Educ.* 28 90–112. 10.1080/08841241.2018.1425231

[B29] Gössling S. Scott D. Michael H. C. (2020). Pandemics, tourism and global change: a rapid assessment of COVID-19. *J. Sustain. Tour.* 29 1–20. 10.1080/09669582.2020.1758708

[B30] Greenhalgh L. Rosenblatt Z. (1984). Job insecurity: toward conceptual clarity. *Acad. Manage. Rev.* 9 438–448. 10.5465/amr.1984.4279673

[B31] Guo S.-L. Lumineau F. Lewicki R. J. (2017). Revisiting the foundations of organizational distrust. *Found. Trends Manage.* 1 1–88. 10.1561/3400000001

[B32] Hair J. F. (2010). *Multivariate Data Analysis.* Hoboken, NJ: Prentice Hall.

[B33] Hair J. F. Hult G. T. M. Ringle C. Sarstedt M. (2016). *A Primer on Partial Least Squares Structural Equation Modeling (PLS-SEM).* Thousand Oaks, CA: SAGE Publications.

[B34] Hair J. F. Sarstedt M. Ringle C. M. Gudergan S. P. (2017). *Advanced Issues in Partial Least Squares Structural Equation Modeling.* Thousand Oaks, CA: SAGE Publications. 10.1007/978-3-319-05542-8_15-1

[B35] Halbesleben J. R. Buckley M. R. (2004). Burnout in organizational life. *J. Manage.* 30 859–879. 10.1016/j.jm.2004.06.004

[B36] Henseler J. Ringle C. M. Sarstedt M. (2015). A new criterion for assessing discriminant validity in variance-based structural equation modeling. *J. Acad. Mark. Sci.* 43 115–135. 10.1007/s11747-014-0403-8

[B37] Hobfoll S. E. (1989). Conservation of resources: a new attempt at conceptualizing stress. *Am. Psychol.* 44 513–524. 10.1037/0003-066X.44.3.513 2648906

[B38] Hobfoll S. E. (2001). The influence of culture, community, and the nested-self in the stress process: advancing conservation of resources theory. *Appl. Psychol.* 50 337–421. 10.1111/1464-0597.00062

[B39] ILO (2020). *COVID-19 and Employment in the Tourism Sector: Impact and Response in Asia and the Pacific.* Geneva: International Labour Organization (ILO).

[B40] Jiang L. Cheng Y. Yang L. Li J. Yan H. Wang X. (2019). A trust-based collaborative filtering algorithm for E-commerce recommendation system. *J. Ambient Intell. Humaniz. Comput.* 10 3023–3034. 10.1007/s12652-018-0928-7

[B41] Kaiser H. F. (1974). An index of factorial simplicity. *Psychometrika* 39 31–36. 10.1007/BF02291575

[B42] Kaiser H. F. Rice J. (1974). Little Jiffy, Mark Iv. *Educ. Psychol. Meas.* 34 111–117. 10.1177/001316447403400115

[B43] Kang H. Y. Kim S. Han K. (2018). The relationship among workplace bullying, organizational commitment and turnover intention of the nurses working in public. *J. Korean Clin. Nurs. Res.* 24 178–187.

[B44] Karatepe O. M. Olugbade O. A. (2017). The effects of work social support and career adaptability on career satisfaction and turnover intentions. *J. Manage. Organ.* 23 337–355. 10.1017/jmo.2016.12

[B45] Karatepe O. M. Rezapouraghdam H. Hassannia R. (2020). Does employee engagement mediate the influence of psychological contract breach on pro environmental behaviors and intent to remain with the organization in the hotel industry? *J. Hosp. Mark. Manage.* 30 326–353. 10.1080/19368623.2020.1812142

[B46] Karatepe O. M. (2013). High-performance work practices, work social support and their effects on job embeddedness and turnover intentions. *Int. J. Contemp. Hosp. Manage.* 25 903–921. 10.1108/IJCHM-06-2012-0097

[B47] Karim M. W. Haque A. Ulfy M. A. Hossain M. A. Anis M. Z. (2020). Factors influencing the use of E-wallet as a payment method among Malaysian young adults. *J. Int. Bus. Manage.* 3 1–12.

[B48] Kessler R. C. Walters E. E. (1998). Epidemiology of DSM-III-R major depression and minor depression among adolescents and young adults in the national comorbidity survey. *Depress. Anxiety* 7 3–14. 10.1002/(SICI)1520-6394(1998)7:1<3::AID-DA2>3.0.CO;2-F 9592628

[B49] Kim D.-K. Yang H.-S. (2017). The effect of organizational justice and psychological contract on turnover intention as mediated by trust and distrust. *J. Digit. Converg.* 15 115–126.

[B50] Lewicki R. J. Bunker B. B. (1996). “Developing and maintaining trust in work relationships,” in *Trust in Organizations: Frontiers of Theory and Research*, eds Kramer R. M. Tyler T. R. (Thousand Oaks, CA: Sage Publications), 114–139. 10.4135/9781452243610.n7

[B51] Li J. J. Wong I. A. Kim W. G. (2016). Effects of psychological contract breach on attitudes and performance: the moderating role of competitive climate. *Int. J. Hosp. Manage.* 55 1–10.

[B52] Li S. Chen Y. (2018). The relationship between psychological contract breach and employees’ counterproductive work behaviors: the mediating effect of organizational cynicism and work alienation. *Front. Psychol.* 9:1273. 10.3389/fpsyg.2018.01273 30100888 PMC6072872

[B53] Lohmöller J. B. (1989). *Latent Variable Path Modeling with Partial Least Squares.* Heidelberg: Physica-Verlag. 10.1007/978-3-642-52512-4

[B54] Loi R. Ao O. K. Xu A. J. (2014). Perceived organizational support and coworker support as antecedents of foreign workers’ voice and psychological stress. *Int. J. Hosp. Manage.* 36 23–30. 10.1016/j.ijhm.2013.08.001

[B55] Maslach C. Schaufeli W. B. Leiter M. P. (2001). Job burnout. *Annu. Rev. Psychol.* 52 397–422. 10.1146/annurev.psych.52.1.397 11148311

[B56] Moore J. E. (2000). Why is this happening? A causal attribution approach to work exhaustion consequences. *Acad. Manage. Rev.* 25 335–349. 10.2307/259017

[B57] Morrison E. W. Robinson S. L. (1997). When employees feel betrayed: a model of how psychological contract violation develops. *Acad. Manage. Rev.* 22 226–256. 10.2307/259230

[B58] Nunnally J. C. (1978). *Psychometric Theory*, 2nd Edn. New York, NY: McGraw Hill.

[B59] Piccoli B. De Witte H. (2015). Job insecurity and emotional exhaustion: testing psychological contract breach versus distributive injustice as indicators of lack of reciprocity. *Work Stress* 29 246–263. 10.1080/02678373.2015.1075624

[B60] Podsakoff P. M. MacKenzie S. B. Lee J.-Y. Podsakoff N. P. (2003). Common method biases in behavioral research: a critical review of the literature and recommended remedies. *J. Appl. Psychol.* 88 879–903. 10.1037/0021-9010.88.5.879 14516251

[B61] Robinson S. L. Morrison E. W. (2000). The development of psychological contract breach and violation: a longitudinal study. *J. Organ. Behav.* 21 525–546. 10.1002/1099-1379(200008)21:5<525::AID-JOB40<3.0.CO;2-T

[B62] Qadri S. U. Bilal M. A. Li M. Ma Z. Qadri S. Ye C. (2022). Work environment as a moderator linking green human resources management strategies with turnover intention of millennials: a study of Malaysian hotel industry. *Sustainability* 14:740. 10.3390/su14127401

[B63] Restubog S. L. D. Bordia P. Tang R. L. (2006). Effects of psychological contract breach on performance of IT employees: the mediating role of affective commitment. *J. Occup. Organ. Psychol.* 79 299–306. 10.1348/096317905X53183

[B64] Russell J. A. Mehrabian A. (1974). Distinguishing anger and anxiety in terms of emotional response factors. *J. Consult. Clin. Psychol.* 42 79–83. 10.1037/h0035915 4814102

[B65] Sarikaya M. Bayrak Kök S. (2017). The relationship between psychological contract breach and organizational cynicism. *Eur. Sci. J.* 13 125–142.

[B66] Sarstedt M. Hair J. F. Ringle C. M. Thiele K. O. Gudergan S. P. (2016). Estimation issues with PLS and CBSEM: where the bias lies! *J. Bus. Res.* 69 3998–4010. 10.1016/j.jbusres.2016.06.007

[B67] Shen Y. Schaubroeck J. M. Zhao L. Wu L. (2019). Work group climate and behavioral responses to psychological contract breach. *Front. Psychol.* 10:67. 10.3389/fpsyg.2019.00067 30778308 PMC6369362

[B68] Staufenbiel T. König C. J. (2010). A model for the effects of job insecurity on performance, turnover intention, and absenteeism. *J. Occup. Organ. Psychol.* 83 101–117. 10.1348/096317908X401912

[B69] Susskind A. M. Kacmar K. M. Borchgrevink C. P. (2003). Customer service providers’ attitudes relating to customer service and customer satisfaction in the customer-server exchange. *J. Appl. Psychol.* 88 179–187. 10.1037/0021-9010.88.1.179 12675405

[B70] Wixom B. H. Watson H. J. (2001). An empirical investigation of the factors affecting data warehousing success. *MIS Q.* 25 17–41. 10.2307/3250957

